# Supplementary material for: Salivary Metabolomics as a Diagnostic Tool: Distinct Metabolic Profiles Across Orofacial Pain Subtypes
Source: Int J Mol Sci. 2025 Mar 3;26(5):2260. doi: 10.3390/ijms26052260 (PMC11900362; doi:10.3390/ijms26052260)
Supplement: Supplementary file 1 [file ijms-26-02260-s001.zip › Supplementary Figures.pdf]

Supplementary Figures

Supplementary Figure S1. Boxplots presenting metabolites showing a significant change in measured intensity for a control group versus the pain subcategories. * *p*-value <0.05; ** *p*-value<0.005; *** *p*-value <0.001; **** *p*-value <0.0001.

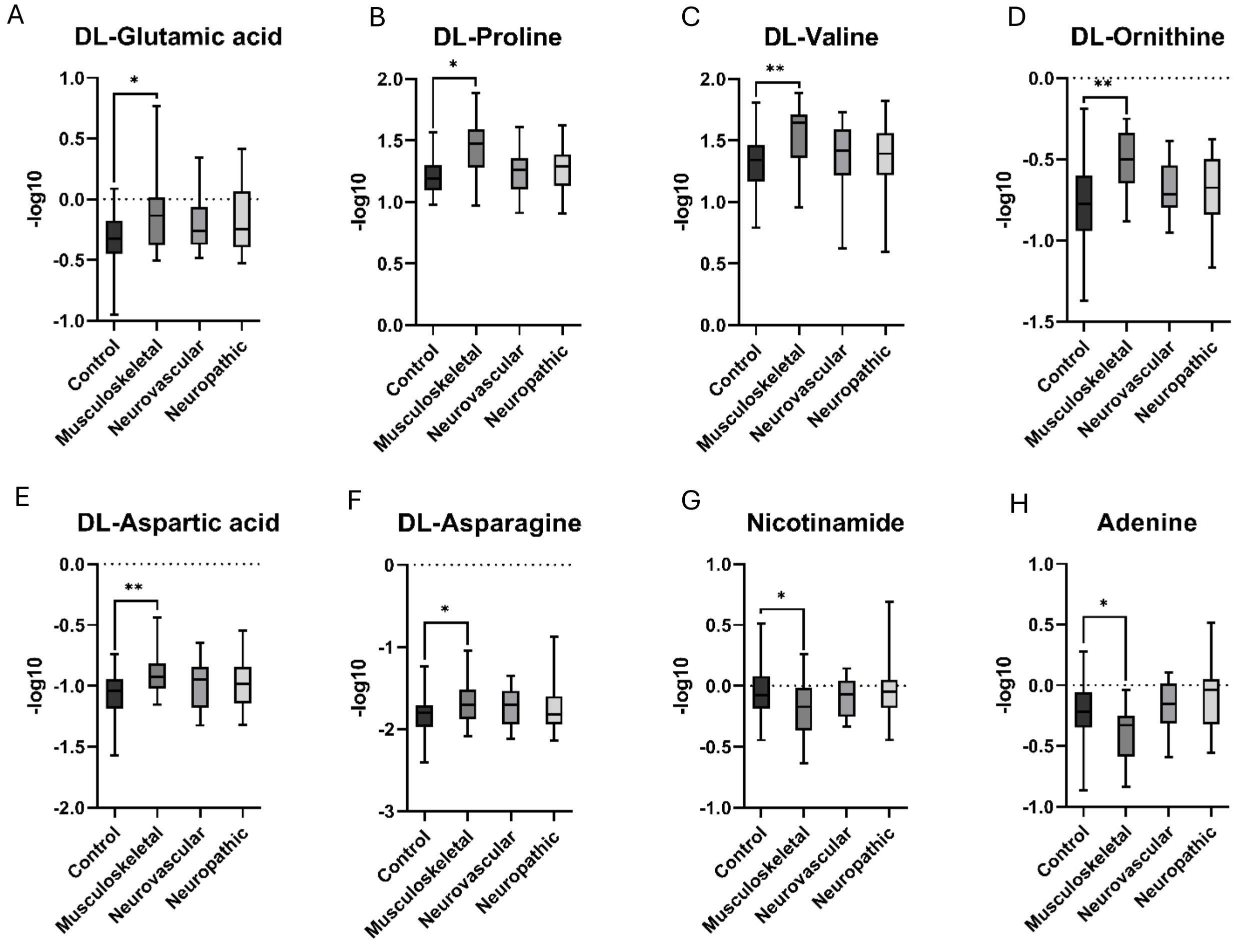

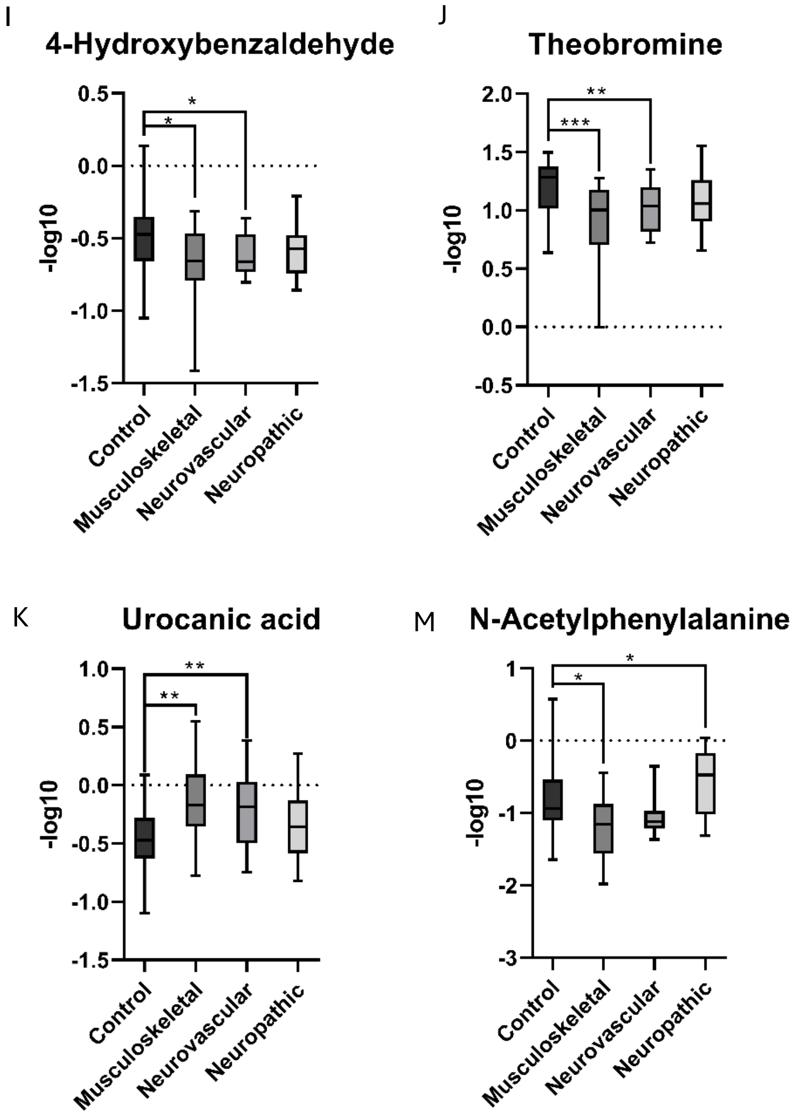
I
4-Hydroxybenzaldehyde
-log10
0.5
0.0
-0.5
-1.0
-1.5
*
*
Control
Musculoskeletal
Neurovascular
Neuropathic
J
Theobromine
-log10
2.0
1.5
1.0
0.5
0.0
-0.5
**
***
Control
Musculoskeletal
Neurovascular
Neuropathic
K
Urocanic acid
-log10
1.0
0.5
0.0
-0.5
-1.0
-1.5
**
**
Control
Musculoskeletal
Neurovascular
Neuropathic
M
N-Acetylphenylalanine
-log10
1
0
-1
-2
-3
*
*
Control
Musculoskeletal
Neurovascular
Neuropathic

Supplementary Figure S2. Boxplots presenting metabolites showing a significant change in measured intensity for a control group versus types of pain. * *p*-value <0.05; ** *p*-value<0.005; *** *p*-value <0.001; **** *p*-value <0.0001.

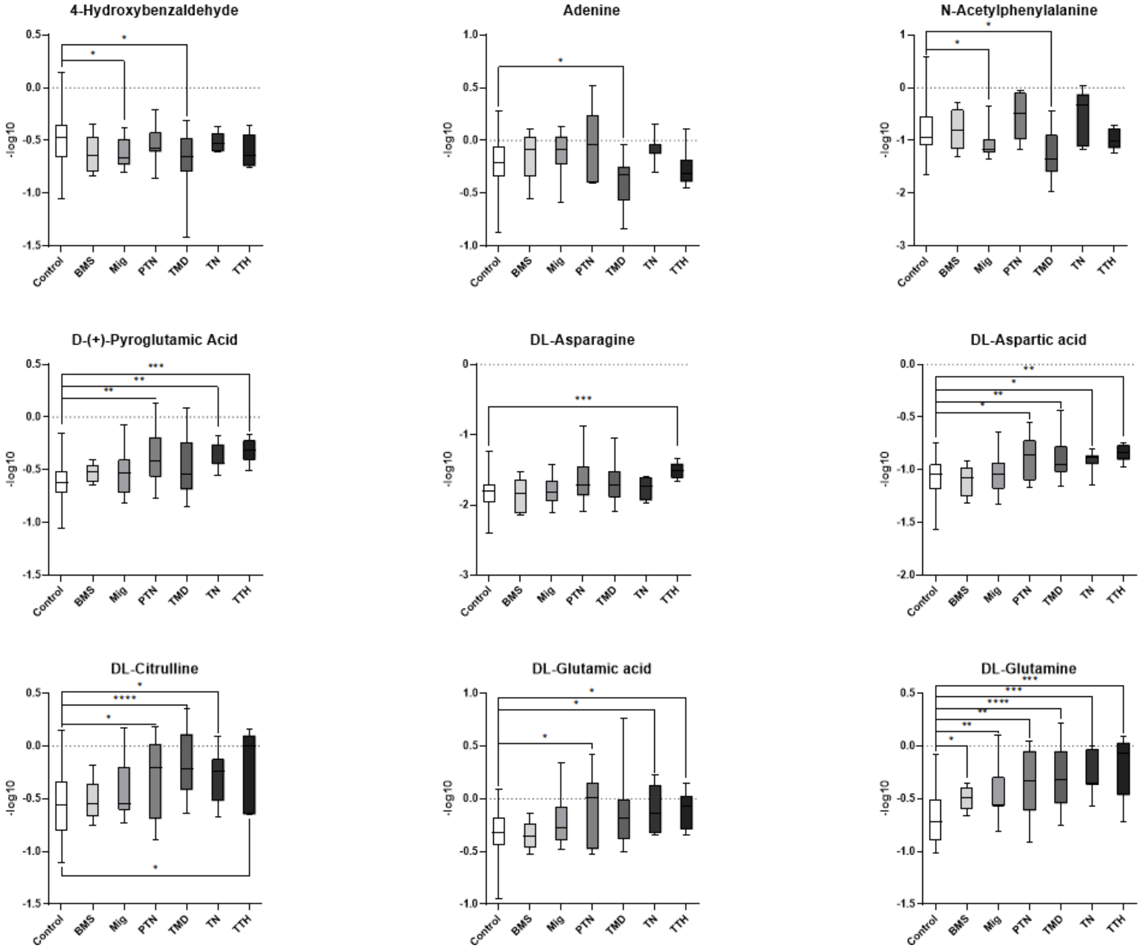

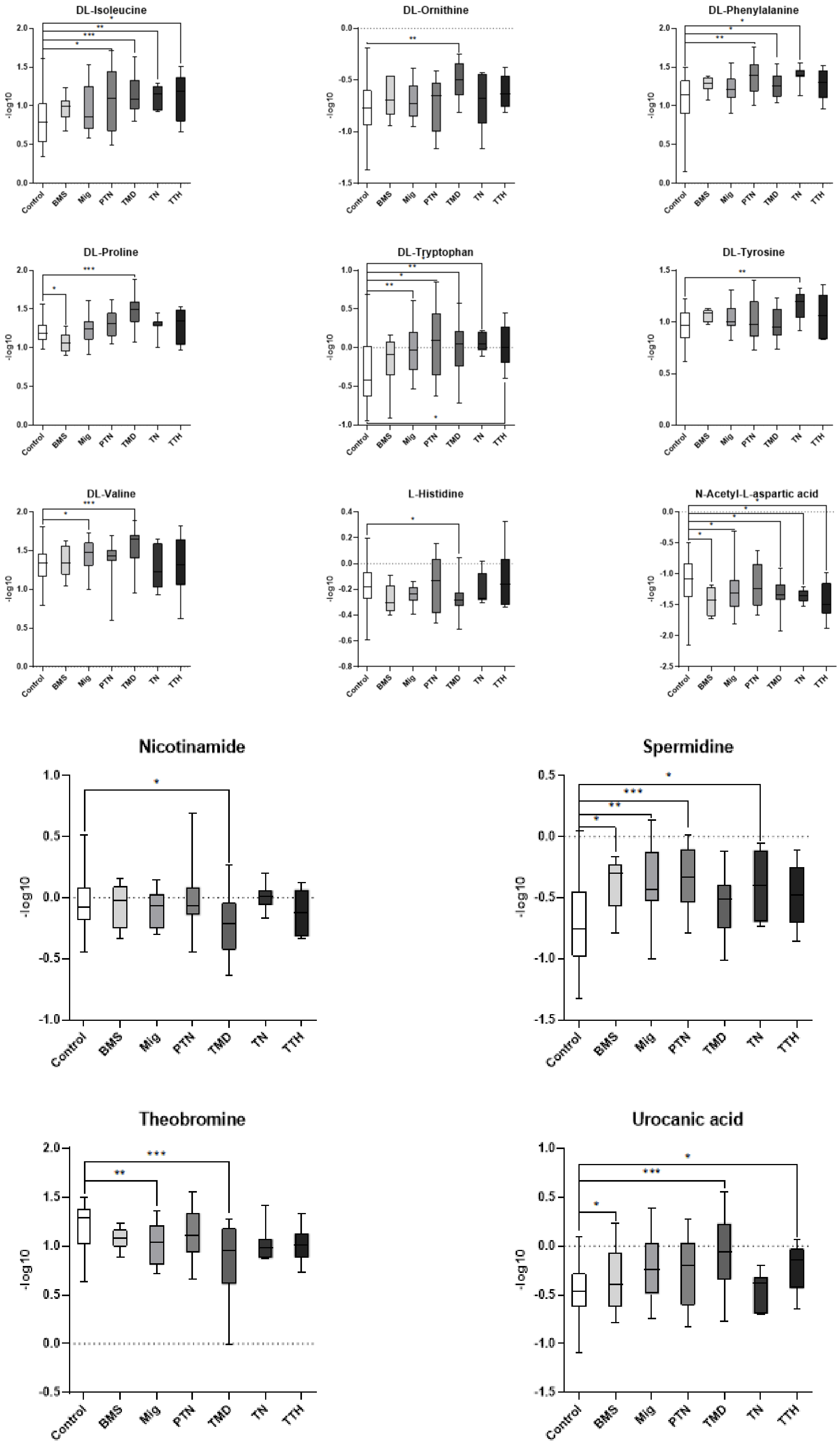
DL-Isoleucine
DL-Ornithine
DL-Phenylalanine
DL-Proline
DL-Tryptophan
DL-Tyrosine
DL-Valine
L-Histidine
N-Acetyl-L-aspartic acid
Nicotinamide
Spermidine
Theobromine
Urocanic acid
-log10
Control
BMS
Mig
PTN
TMD
TN
TTH
